# Artificial intelligence performance in detecting lymphoma from medical imaging: a systematic review and meta-analysis

**DOI:** 10.1186/s12911-023-02397-9

**Published:** 2024-01-08

**Authors:** Anying Bai, Mingyu Si, Peng Xue, Yimin Qu, Yu Jiang

**Affiliations:** 1 School of Population Medicine and Public Health, Chinese Academy of Medical Sciences and Peking Union Medical College, Beijing, China; 2 School of Health Policy and Management, Chinese Academy of Medical Sciences and Peking Union Medical College, Beijing, China

**Keywords:** Diagnosis, Artificial intelligence, Lymphoma, Medical imaging, Meta

## Abstract

**Background:**

Accurate diagnosis and early treatment are essential in the fight against lymphatic cancer. The application of artificial intelligence (AI) in the field of medical imaging shows great potential, but the diagnostic accuracy of lymphoma is unclear. This study was done to systematically review and meta-analyse researches concerning the diagnostic performance of AI in detecting lymphoma using medical imaging for the first time.

**Methods:**

Searches were conducted in Medline, Embase, IEEE and Cochrane up to December 2023. Data extraction and assessment of the included study quality were independently conducted by two investigators. Studies that reported the diagnostic performance of an AI model/s for the early detection of lymphoma using medical imaging were included in the systemic review. We extracted the binary diagnostic accuracy data to obtain the outcomes of interest: sensitivity (SE), specificity (SP), and Area Under the Curve (AUC). The study was registered with the PROSPERO, CRD42022383386.

**Results:**

Thirty studies were included in the systematic review, sixteen of which were meta-analyzed with a pooled sensitivity of 87% (95%CI 83–91%), specificity of 94% (92–96%), and AUC of 97% (95–98%). Satisfactory diagnostic performance was observed in subgroup analyses based on algorithms types (machine learning versus deep learning, and whether transfer learning was applied), sample size (≤ 200 or >  200), clinicians versus AI models and geographical distribution of institutions (Asia versus non-Asia).

**Conclusions:**

Even if possible overestimation and further studies with a better standards for application of AI algorithms in lymphoma detection are needed, we suggest the AI may be useful in lymphoma diagnosis.

**Supplementary Information:**

The online version contains supplementary material available at 10.1186/s12911-023-02397-9.

## Introduction

As a clonal malignancy of lymphocytes, lymphoma are diagnosed in 280,000 people annually worldwide with divergent patterns of clinical behavior and responses to treatment [1]. Based on the WHO classification, non-Hodgkin lymphoma (NHL) derived from mature lymphoid cells brings about 6,991,329 (90.36%) disability-adjusted life-years (DALYs), and Hodgkin lymphoma (HL) originated from precursor lymphoid cells accounts for 14.81% of DALYs [2, 3]. Since about 30% cases of NHL arise in extranodal sites [4], some are considered very aggressive (i.e., Diffuse large B-cell lymphoma in NHL). Early and timely detection of lymphoma are needed to forward the qualified treatment and improve the post-operative quality of life.

Since lymphocyte had diverse physiologic immune function according to lineage and differentiation stage, the classification of lymphomas arising from these normal lymphoid populations is complicated. Imaging is a useful tool in medical science and is invoked in clinical practice to facilitate decision making for the diagnosis, staging, and treatment [5]. Despite advances in medical imaging technology, it is difficult for even experienced hematopathologists to identify different subtypes of lymphoma. Diagnosis of lymphoma is firstly based on the pattern of growth and the cytologic features of the abnormal cells, then clinical, molecular pathology, immunohistochemical, and genomic features are required to finalize the identification of certain subtypes [6]. However, clinical routine methods that enable tissue-specific diagnosis, such as image-guided tumor biopsy and percutaneous needle aspiration, have the shortcomings of subjectivity, costly, and poor classification accuracy [7]. Diagnostic features vary widely (from 14.8 to 27.3%) due to inter-observer variability among experts using multiple imaging methods such as computed tomography (CT), magnetic resonance imaging (MRI), and Whole Slide Image (WSI) in the same sample [8]. As diagnostic accuracy of lymphoma depends largely on the clinical judgment of physicians and the technical process of tissue sections, limited health system capacities and competing health priorities in more resource-deprived areas may lack infrastructure and perhaps the manpower to ensure high-quality detection of lymphoma. Therefore, accurate, objective and cost-effective methods are required for the early diagnosis of lymphoma in clinical settings and ultimately provide better guidance for lymphoma therapies.

Artificial intelligence (AI) offers tremendous opportunities in this field. It has the ability to extend the noninvasive study of oncologic tissue beyond established imaging metrics, to assist automatic image classification, and to facilitate performance of cancer diagnosis [9–11]. As branches of AI, machine learning (ML) [12, 13] and deep learning (DL) [8, 14] have shown promising results for detection of malignant lymphoma. However, there are no studies systematically assessing the diagnostic performance of AI algorithms in identifying lymphoma. Here, we performed a meta-analysis to assess the diagnostic accuracy of AI algorithms that use medical imaging to detect lymphoma.

## Materials and methods

The study protocol was approved on the PROSPERO (CRD42022383386). This meta-analysis was conducted according to the Preferred Reporting Items for Systematic reviews and Meta-analyses (PRISMA) 2020 guidelines [15]. Ethical approval was not applicable.

### Search strategy and eligibility criteria

In this study, we searched Medline, Embase, IEEE and the Cochrane library until December 2023. No restrictions were applied around regions, languages, participant characteristics, type of imaging modality, AI models or publication types. The full search strategy was developed in collaboration with a group of experienced clinicians and medical researchers (see Additional file 1).

Eligibility assessment was conducted independently by two investigators, who screened titles and abstracts, and selected all relevant citations for full-text review. Disagreements were resolved through discussion with another collaborator. We included all published studies that reported the diagnostic performance of a AI model/s for the early detection of lymphoma using medical imaging. Studies that met the following criteria were included in the final group: (1) Any study that analyzed medical imaging for diagnosis of lymphoma with AI-based models; (2) Studies that provided any raw diagnostic performance data, such as sensitivity, specificity, area under curve (AUC) accuracy, negative predictive values (NPVs), or positive predictive values (PPVs). The primary outcomes were diagnostic performance indicators. Studies were excluded when they met the following criteria: (1) Case reports, review articles, editorials, letters, comments, and conference abstracts; (2) Studies that used medical waveform data graphics material (i.e., electroencephalography, electrocardiography, and visual field data) or investigated the accuracy of image segmentation rather than disease classification; (3) Studies without the outcome of disease classification or not target diseases; (4) Studies that did not use histopathology and expert consensus as the study reference standard of lymphoma diagnosis; (5) Studies that use animals’ studies or non-human samples; (6) Duplicate studies.

### Data extraction

Two investigators independently extracted study characteristics and diagnostic performance data using a predetermined data extraction sheet. Again, uncertainties were resolved by a third investigator. Where possible, we extracted binary diagnostic accuracy data and constructed contingency tables at the reported thresholds. Contingency tables contained true-positive (TP), false-positive (FP), true-negative (TN), and false-negative (FN) values and were used to determine sensitivity and specificity. If a study provided multiple contingency tables for the same or for different AI algorithms, we assumed that they were independent of each other.

### Quality assessment

The quality assessment of diagnostic accuracy studies-AI (QUADAS-AI) criteria was used to assess the risk of bias and applicability concerns of the included studies [16], which is an AI-specific extension to QUADAS-2 [17] and QUADAS-C [18].

### Meta-analysis

Hierarchical summary receiver operating characteristic (SROC) curves were used to assess the diagnostic performance of AI algorithms. Hierarchical SROC provided more credibility to the analysis of small sample size, taking both between and within study variation into account. 95% confidence intervals (*CI*) and prediction regions were generated around averaged sensitivity, specificity, and AUCs estimates in Hierarchical SROC figures. Heterogeneity was assessed using the *I*^*2*^ statistic. We performed subgroup and regression analyses to explore the potential effects of different sample size (≤200 or >  200), diagnostic performance using the same dataset (AI algorithms or human clinicians), AI algorithms (ML or DL), geographical distribution (Asia or non-Asia), and application of transfer learning (Yes or No). The random effects model was implemented since the assumed differences between studies. The risk of publication bias was assessed using funnel plot.

We evaluated the quality of included studies by RevMan (Version 5.3). A cross-hairs plot was produced (R V.4.2.1) to better display the variability between sensitivity/specificity estimates. All other statistical analyses were conducted using Stata (Version 16.0). Two-sided *p* < 0.05 was the threshold for statistical significance.

## Results

### Study selection and characteristics

**Fig. 1:**
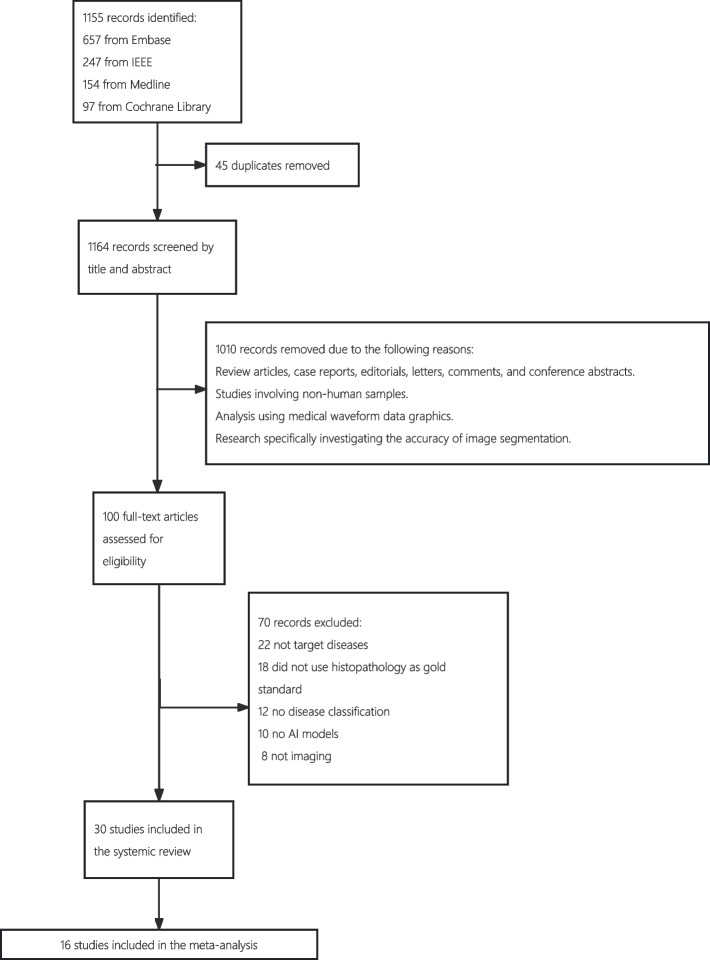
PRISMA flow chart outlining the selection of studies for review

Our search initially identified 1155 records, of which 1110 were screened after removing 45 duplicates. 1010 were also excluded as they did not fulfill our predetermined inclusion criteria. A total of 100 full-text articles were reviewed, 70 were excluded, and the remaining 30 focused on lymphomas (see Fig. 1) [1, 8, 12–14, 19–43]. Study characteristics are summarized in Tables 1, 2 and 3.

**Table 1:** Participant demographics for the 24 included studies

First author and year	Participants			
	Inclusion criteria	Exclusion criteria	*N*	Mean age (SD; range, year)
Zhou Z, 2021	Patients with biopsy-confirmed MCL from May 2007 to October 2018.	The PET and CT slice numbers were different; either the PET or CT images could not be extracted from the picture archiving and communication system; the reference standard contour cannot be established.	142	Within-institution: 58 (NR; 39–84), outside-institution: 59 (NR; 40–67)
McAvoy M, 2021	Age ≥ 18 years old; pathology confirmed diagnoses of GBM or PCNSL that was untreated (i.e. not recurrent).	Patients with incomplete MR scans or scans with movement artifacts.	320	Training group: 63.9 (NR; 20–89), Testing group: 62.9 (NR; 40–83)
Li D, 2020	DLBCL patients from three independent hospitals.	NR	867	NR
Miyoshi H, 2020	DLBCL, FL or RL diagnosed at Kurume University from 2010 to 2017.	NR	388	NR
Park JE, 2020	Patients under 19 years of age who underwent neck US with pathologic confirmation of the lymphadenopathy from 2012 to 2018.	Disease groups with less than twenty patients and inconclusive pathologic results.	242	11.2 (0.3; 1 month to 18)
Mohlman JS, 2020	Patients with quality preserved H&E slides of BL and DLBCL.	Other subtypes/variants such as T-cell/histiocyte rich large B-cell lymphoma.	70	NR
Achi HE, 2019	NR	NR	128	NR
Im H, 2018	NR	NR	40	NR
Guan Q, 2019	NR	NR	80	NR
Guo R, 2021	Patients with histopathologically diagnosed ENKTL.	Patients who had undergone surgical resection, radiotherapy, chemotherapy, and/or bone marrow transplantation as well as those with other malignancies.	167	NR
Xia W, 2021	PCNSL was proven by histopathology; underwent preoperative MRI.	Patients lacking any one of the MRI sequences; the images contained serious artifacts.	289	54 (13; 16–82)
Zhang Y, 2021	PCNSL confirmed by pathology; available cerebral MRI before diagnosis.	Patient age < 18 years; missing clinical information; receipt of hormone therapy before undergoing MRI; no data on enhanced MRI; lesions not in the cerebral parenchyma; and MR images with obvious artifact.	92	53.34 (12.57; NR)
Syrykh C, 2020	NR	NR	NR	NR
Wang H, 2020	Pathologically diagnosed ENKTL between January 2011 and January 2017, pretreatment 18F-FDG PET/CT.	NR	110	45.59 (14.55; NR)
Zhang J, 2020	NHL confirmed by histopathology.	NR	374	NR
Wang Q, 2017	Patients having been clinically examined at the hospital where the study was conducted.	NR	27	NR (NR; 7–65)
Schouten JPE, 2021	NA	Duplicated images.	250	NR
Nakagawa M, 2018	Patients with intraparenchymal brain tumor pathologically proven as GBM or PCNSL; the contrast-enhanced MRI exam were performed within 1 month before surgery.	Patients had neither pathologically proven GBM nor PCNSL; patient performed with other type of scanner; patients without a perfusion study; poor image quality.	70	69 (13; 26–88)
Shafique S, 2018	NR	NR	368	NR
Kong Z, 2019	Age ≥ 18 years old; underwent surgical resection or biopsy with pathology confirmed primary CNS lymphoma or GBM; had a preoperative 18F-FDG-PET/CT scan of the brain.	A history of brain tumors; suspected or confirmed peripheral lymphomas; treated with corticosteroids, radiotherapy or chemotherapy before surgery; diabetes mellitus (blood glucose ≥10 mmol/L); iatrogenic or disease-related immunosuppression.	77	58.83 (12.67; NR)
Weisman AJ, 2020	NR	NR	90	Hutchings: 38 (NR; 19–66), Denmark: 37(NR; 16–76), Mylam: 61 (NR; 23–83)
Kim Y, 2018	Pathologically confirmed glioblastoma or PCNSL; no prior biopsy or treatment; and adequate image quality without artifacts.	Patients without pretreatment MRI, high-resolution CE-T1-weighted image, or DWI; poor image quality.	64	59 (13.6; 20–80)
Styczeń M, 2012	NR	NR	30	64.5 (14.4; 28–84)
Guo J, 2018	Histopathologically confirmed cases of primary OAL or IOI; patients with orbital MRI (including pre- and post-contrast studies) less than 14 days before biopsy or surgery; patients with no history of surgery or treatment in the affected orbits.	Poor image quality; orbital lesions less than 0.5 cm in short diameter; OAL secondary to systemic lymphoma; patients with IOI or OAL.	84	Test set: 50.55 (14.03; 5–85), validation set: 55.37 (13.36; 25–80)
Azamossadat H, 2023	NR	NR	89	NR
Chava P, 2023	Patients who were analyzed through FISH as part of their pathological workup	Patients with non-informative FISH results, attributed to technical issues	55	Entire Cohort (62; 8–84) Training set (66.5; 8–84) Validation set (60; 17–77)
Jermphiphut J, 2023	Patients with PCNSL or GBM who had confirmed diagnoses from tissue specimens by a pathologist between January 2010 and December 2021.	Patients were ex- cluded as follows: (1) patients with missing MRI scans; (2) patients with inadequate MRI with move- ment artifacts.	274	54.1 (14.1)
Hikaru A, 2023	Ppatients pathologically confirmed with diffuse large B-cell lymphoma who underwent whole-body [18F] FDG PET/CT imaging before treatment from August 2005 to June.	Patients with comorbid diseases, including known malignancy or active infection, and were under the age of 18 were excluded.	62	64.7 ± 14.0
Manjit K, 2023	NR	NR	20,000	NR
Noriaki H, 2023	NR	NR	249	NR

*NR* not reported, *MCL* mantle cell lymphoma, *GBM* glioblastoma multiforme, *PCNSL* primary central nervous system lymphoma, *DLBCL* diffuse large B-cell lymphoma, *FL* follicular lymphoma, *RL* reactive lymphoid hyperplasia, *DWI* diffusion-weighted imaging, *OAL* ocular adnexal lymphoma, *IOI* idiopathic orbital inflammation, *BL* burkitt lymphoma, *MRI* magnetic resonance imaging, *MR* magnetic resonance

**Table 2:** Model training and validation for the 24 included studies

First author and year	Target condition	Reference standard	Type of internal validation	External validation
Zhou Z, 2021	MCL	Histopathology	Five-fold cross validation	Yes
McAvoy M, 2021	PCNSL	Histopathology	NR	No
Li D, 2020	DLBCL	Histopathology	NR	No
Miyoshi H, 2020	DLBCL, FL	Histopathology or expert consensus	Five-fold cross-validation	No
Park JE, 2020	Lymphoma	Histopathology	NR	Yes
Mohlman JS, 2020	BL, DLBCL	Histopathology	Leave-one-out cross-validation	No
Achi HE, 2019	DLBCL, BL, SLL	Histopathology	NR	No
Im H, 2018	Lymphoma	Histopathology	Random split-sample validation	No
Guan Q, 2019	NHL	Histopathology	NR	No
Guo R, 2021	ENKTL	Histopathology	NR	No
Xia W, 2021	PCNSL	Histopathology	Five-fold cross validation	No
Zhang Y, 2021	PCNSL	Histopathology	NR	No
Syrykh C, 2020	FL	Histopathology	NR	Yes
Wang H, 2020	ENKTL	Histopathology	Ten-fold cross-validation	No
Zhang J, 2020	NHL	NR	Five-fold cross validation	No
Wang Q, 2017	ALL	Histopathology	Cross validation	No
Schouten JPE, 2021	ALL	Expert consensus	Tenfold cross-validation	No
Nakagawa M, 2018	PCNSL	Expert consensus	Ten-fold cross-validation	No
Shafique S, 2018	ALL	Expert consensus	NR	No
Kong Z, 2019	PCNSL	Histopathology	Five-fold cross validation	No
Weisman AJ, 2020	Lymphoma	Expert consensus	Five-fold cross validation	No
Kim Y, 2018	PCNSL	Histopathology	Ten-fold cross-validation	Yes
Styczeń M, 2012	Splenic and gastric marginal zone lymphoma	Histopathology	NR	No
Guo J, 2018	OAL	Histopathology	NR	No
Azamossadat H, 2023	B-ALL	Histopathology	NR	No
Chava P, 2023	DLBCL, HGL	Histopathology	NR	Yes
Jermphiphut J, 2023	PCNSL	Histopathology	NR	No
Hikaru A, 2023	DLBCL	Histopathology	Five-fold cross-validation	No
Manjit K, 2023	ALL	Histopathology	NR	No
Noriaki H, 2023	DLBCL, FL and RL	Histopathology	Five-fold cross-validation	Yes

*NR* not reported, *MCL* mantle cell lymphoma, *PCNSL* primary central nervous system lymphoma, *DLBCL* diffuse large B-cell lymphoma, *HGL* high grade lymphomas, *FL* follicular lymphoma, *BL* burkitt lymphoma, *SLL* small lymphocytic lymphoma, *ENKTL* nasal-type extranodal natural killer/T cell lymphoma, *NHL* non-Hodgkin’s lymphoma, *ALL* acute lymphoblastic leukemia, *OAL* ocular adnexal lymphoma, *RL* reactive lymphoid hyperplasia

**Table 3:** Indicator, algorithm, and data source for the included studies

First author and year	Indicator definition			Algorithm		Data source			
	Method for predictor	Exclusion of poor quality imaging	Heatmap provided	Algorithm architecture	Transfer learning applied	Source of data	Number of images for training/internal/external	Data range	Open access data
Zhou Z, 2021	PET/CT images	N/A	N/A	DLCNN	Yes	Retrospective, data from 33 centers.	110/110/32	2007/05–2018/10	No
McAvoy M, 2021	MRI	Yes	N/A	CNN, EfficientNet	Yes	Retrospective, data from the Partners Healthcare Research Patient Data Registry (RPDR) web-based query tool.	1245/202/NR	2015–2018	No
Li D, 2020	Histopathology images	N/A	N/A	GOTDP-MP-CNNs	Yes	Retrospective, data from 3 hospitals.	80%/10%/NR	NR	Yes
Miyoshi H, 2020	WSI	N/A	N/A	DCNN	No	Retrospective, data from Kurume University.	388/NR/NR	2010–2017	NR
Park JE, 2020	Ultrasound	N/A	N/A	CART	No	Retrospective, data from a single center in US.	170/NR/72	2012–2018	NR
Mohlman JS, 2020	Histologic images	Yes	N/A	Deep dense CNN	Yes	Retrospective, data from hematopathology archives at author’s institutions.	8796/NR/NR	2010–2020	NR
Achi HE, 2019	WSI	N/A	N/A	DLCNN	No	Retrospective, data from Virtual Pathology at the University of Leeds and Virtual Slide Box from University of Iowa.	1856/464/NR	NR	NR
Im H, 2018	Holograms	N/A	N/A	CNN	No	Prospective.	3447/1723/NR	NR	Yes
Guan Q, 2019	Cytological images	No	No	DCNN, Inception-v3	Yes	Retrospective, data from a single center.	156/26/NR	2016.11–2017.11	NR
Guo R, 2021	PET/CT images	No	No	ResNet-18	No	Retrospective and prospective, data from Shanghai Ruijin Hospital.	64/20/NR	2011/06–2020/10	Yes
Xia W, 2021	MRI	N/A	N/A	IF-CNN	Yes	Retrospective, data from a single center.	80%/20%/NR	2011–2019	NR
Zhang Y, 2021	MRI	Yes	No	3D U-Net/Resnet18	No	Retrospective, data from two centers in China.	65/27/NR	2005.1–2019.12	Yes
Syrykh C, 2020	WSI	N/A	N/A	Stochastic BNN	No	Retrospective, data from the lymphopath database in Toulouse University Cancer Institute and Dijon University Hospital, France.	50%/25%/25%	NR	No
Wang H, 2020	PET/CT images	No	No	LASSO	No	Retrospective.	82/28/NR	2011/01–2017/01	NR
Zhang J, 2020	Histopathology images	No	N/A	VGG-16, VGG-19, ResNet-50, DenseNet-121	Yes	Retrospective, data from a reference dataset provided by Janowczyk et al.	269/30/NR	NR	NR
Wang Q, 2017	Microscopic blood images	No	No	ANN	No	Retrospective, data from the Department of Hematology, Ruijin Hospital, Shanghai, China.	27/NR/NR	NR	NR
Schouten JPE, 2021	Cytological images	No	No	CNN	No	Retrospective, data from the Department of Information Technology at the University of Milan.	200/25/25	NR	NR
Nakagawa M, 2018	MRI	N/A	N/A	Machine learning classifiers	No	Retrospective.	63/7/NR	2008.01–2013.12	NR
Shafique S, 2018	Microscopic blood images	No	No	DCNN	Yes	Retrospective, data from ALL-Image DataBase (IDB).	60%/40%/NR	NR	Yes
Kong Z, 2019	PET/CT images	N/A	Yes	Decision tree classifier	No	Retrospective, data from Peking Union Medical College Hospital.	80%/20%/NR	2010/01–2018/10	NR
Weisman AJ, 2020	PET/CT images	No	No	CNN	No	Retrospective, data from one of two separate prospective multicenter imaging trials.	65%/15%/20%	2005–2011	NR
Kim Y, 2018	mpMRI	Yes	No	Logistic regression-based classifier, random forest	No	Retrospective, data from two independent cohorts: the discovery cohort from Samsung Medical Center in Seoul, South Korea; and the validation cohort from Asan Medical Center in Seoul, South Korea.	36/36/28	2013/01–2016/02	NR
Styczeń M, 2012	Specimens	No	No	Neural network	No	Retrospective, data from the Department of Pathology.	NR/NR/NR	2005–2009	NR
Guo J, 2018	MRI	Yes	No	LASSO	No	Retrospective, data from a single center.	47/37/NR	2010/03–2016/07	NR
Azamossadat H, 2023	Microscopic blood images	No	No	CNN	Yes	Restrospective, data from several Tehran hospitals	85/5/10	NR	NR
Chava P, 2023	Biopsy slides	No	No	MIL	No	Restrospective, data from the Tel Aviv Sourasky Medical Center	55/NR/25	2017/01–2022/01	NR
Jermphiphut J, 2023	MRI	No	No	CNN	No	Restrospective, data from a historical cohort study	709/177/NR	2010/01–2021/12	NR
Hikaru A, 2023	PET/CT images	No	Yes	CNN	No	Restrospective, data from Nthe Tokyo Medical and Dental University, Tokyo, Japan, and Shinjuku Tsurukame Clinic, Tokyo, Japan.	62/NR/NR	2005/08–06/2020	NR
Manjit K, 2023	Peripheral blood smear images	No	No	DSCNet	No	Restrospective, data from Acute Lymphoblastic Leukemia Image Dataset	20,000/NR/NR	2021	Yes
Noriaki H, 2023	WSI	No	No	ResNet50	No	Restrospective, data from Kurume University and Nagoya University Hospital	249/NR/208	2018	NR

*NR* not reported, *MRI* magnetic resonance imaging, *mpMRI* multi-parametric MRI, *WSI* whole slide image, *CNN* convolutional neural network, *DCNN* deep convolutional neural network, *IF-CNN* image-level fusion based multi-parametric CNN, *CART* classification and regression tree, *DLCNN* deep learning convolutional neural network, *ResNet* residual network, *BNN* bayesian neural network, *LASSO* least absolute shrinkage and selection operator, *ANN* artificial neural network, *DSCNet* deep skip connections-based dense network, *MIL* multiple instance learning algorithms

Twenty-nine studies utilized retrospective data. Only one study used prospective data. Six studies used data from open access sources. Five studies excluded low-quality images, while ten studies did not report anything about image quality. Six studies performed external validation using the out-of-sample dataset, fifteen studies did not report type of internal validation while the others performed internal validation using the in-sample dataset. Seven studies utilized ML algorithms and twenty-three studies used DL algorithms to detect lymphoma. Three studies compared AI algorithms against human clinicians using the same dataset. Among the studies analyzed, six utilized samples diagnosed with PCNSL, six involved samples with DCBCL, four studies focused on ALL, while two studies focused on NHL. Additionally, individual studies were conducted among patients with ENKTL, splenic and gastric marginal zone lymphomas, and ocular adnexal lymphoma. Furthermore, a variety of medical imaging modalities were employed across the studies: six studies utilized MRI, four used WSI instruments, four employed microscopic blood images, three utilized PET/CT, and two relied on histopathology images.

### Pooled performance of AI algorithms

**Fig. 2:**
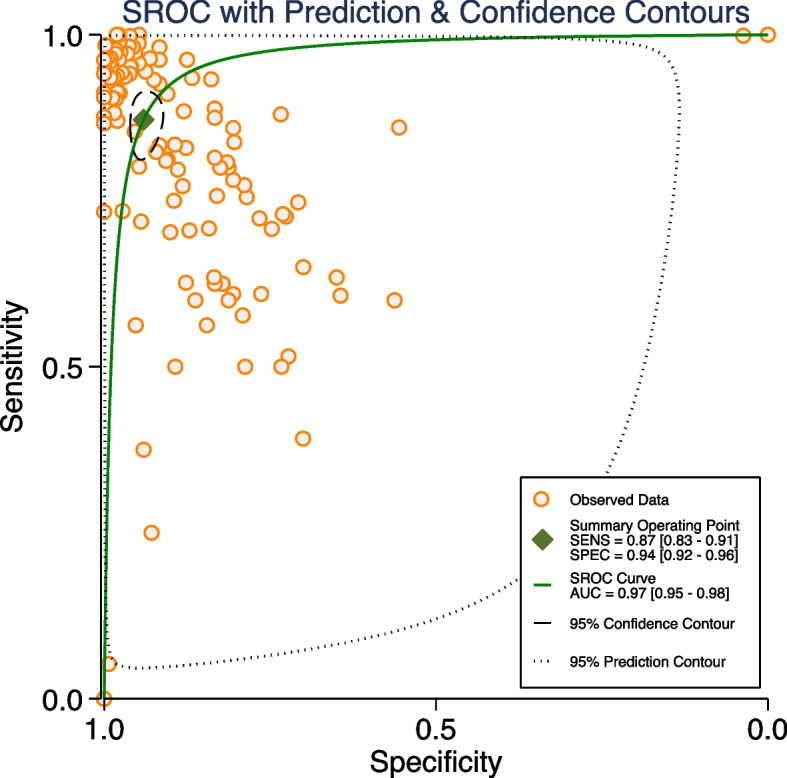
Hierarchical SROC curves for studies included in the meta-analysis (16 studies with 124 tables)

Among the included 30 studies, 16 provided enough data to assess diagnostic performance and were thus included in the meta-analysis [1, 12, 14, 20, 22–26, 28, 29, 32, 33, 35–37]. Hierarchical SROC curves for these studies are provided in Fig. 2. When averaging across studies, the pooled SE and SP were 87% (95% CI 83–91%), and 94% (95% CI 92–96%), respectively, with an AUC of 0.97 (95% CI 0.95–0.98) for all AI algorithms.

### Heterogeneity analysis

**Fig. 3:**
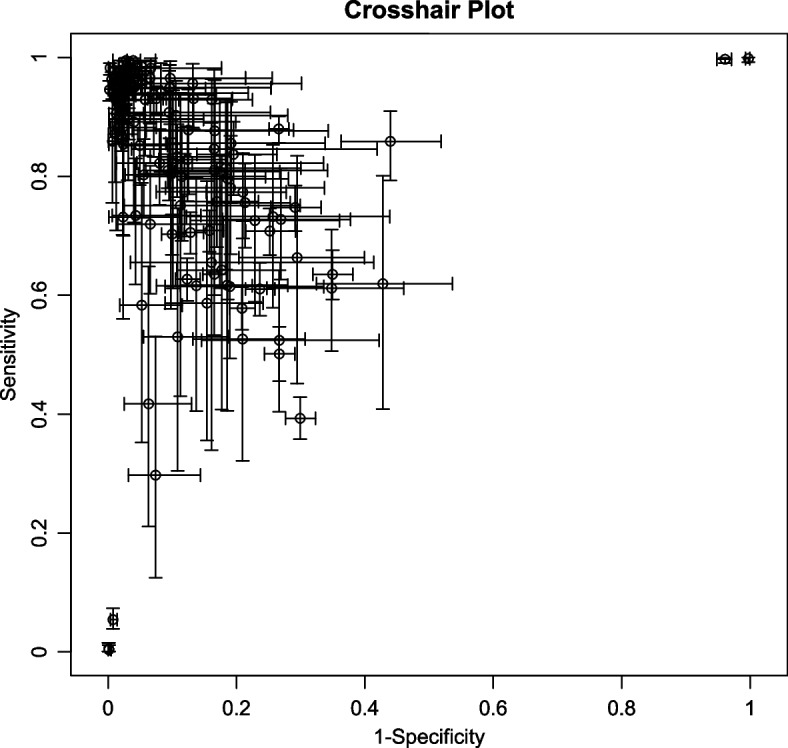
Cross-hair Plot of studies included in the meta-analysis (16 studies with 124 tables)

**Fig. 4:**
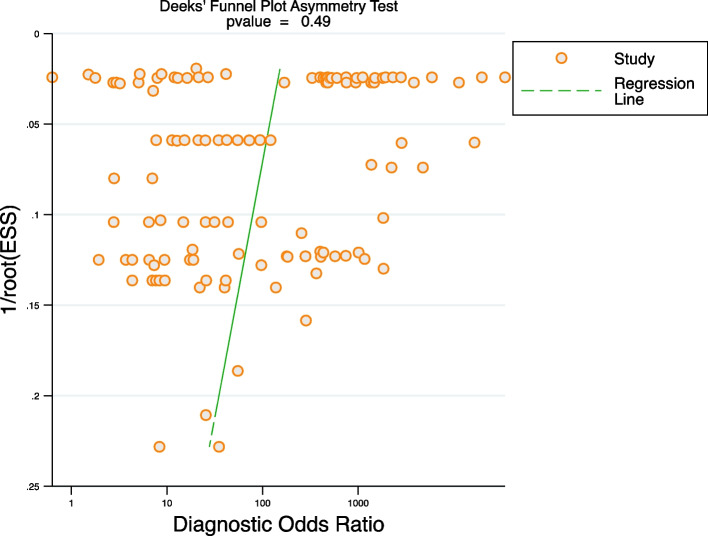
Deeks’ Funnel Plot Asymmetry Test

All included studies found that AI algorithms were useful for the detection of lymphoma using medical imaging when compared with reference standards; however, extreme heterogeneity was observed. Sensitivity (SE) had an *I*^*2*^ = 99.35%, while specificity (SP) had an *I*^*2*^ = 99.68% (*p* < 0.0001), see Fig. 3. The detailed results of subgroup and meta-regression analyses are shown in Table 4. The heterogeneity for the pooled specificity and sensitivity are still significant within each subgroup, suggesting potential sources of inter-study heterogeneity among studies with different sample sizes, various algorithms applied, geographical distribution and Al algorithms-assisted clinicians versus pure clinicians. However, the results of meta-regression highlight that only difference in AI algorithms and human clinicians remain statistically significant, indicating a potential source of between-subgroup heterogeneity. Furthermore, a funnel plot was produced to assess publication bias, see Fig. 4. The *p* value of 0.49 suggests there is no publication bias although studies were widely dispersed around the regression line.

**Table 4:** Summary estimate of pooled performance of artificial intelligence in lymphoma detection

	No. of studies				*P* value^b^				*P* value
Overall	16	Sensitivity	*P* value ^a^	I^2^(95%CI)		Specificity	*P* value	I^2^ (95%CI)	
Algorithm					0.11				0.83
Deep Learning	13	0.86 (0.80–0.90)	< 0.05	99.41 (99.37–99.47)		0.94 (0.92–0.96)	< 0.05	99.71 (99.70–99.72)	
Machine Learning	3	0.93 (0.88–0.95)	< 0.05	91.47 (88.74–94.21)		0.92 (0.87–0.95)	< 0.05	87.72 (83.33–92.10)	
Transfer Learning Applied					0.92				0.55
Yes	6	0.88 (0.80–0.93)	< 0.05	99.67 (99.65–99.69)		0.95 (0.92–0.97)	< 0.05	99.85 (99.84–99.85)	
No	10	0.85 (0.80–0.89)	< 0.05	91.29 (89.67–92.91)		0.91 (0.88–0.93)	< 0.05	92.39 (91.04–93.75)	
Human Clinicians versus Algorithms				0.01				< 0.05	
Clinicians	3	0.70 (0.65–0.75)	< 0.05	77.53 (69.54–85.53)		0.86 (0.82–0.89)	< 0.05	84.09 (78.94–89.23)	
Algorithms	13	0.91 (0.86–0.94)	< 0.05	99.60 (99.58–99.62)		0.96 (0.93–0.97)	< 0.05	99.81 (99.80–99.82)	
Sample size					0.45				0.39
≤ 200	11	0.88 (0.84–0.92)	< 0.05	98.71 (98.55–98.86)		0.91 (0.87–0.94)	< 0.05	99.02 (98.91–99.13)	
> 200	5	0.86 (0.78–0.91)	< 0.05	99.47 (99.43–99.50)		0.95 (0.92–0.97)	< 0.05	99.77 (99.76–99.78)	
Geographical distribution					0.67				0.51
Asia	10	0.88 (0.83–0.91)	< 0.05	99.34 (99.30–99.38)		0.94 (0.92–0.96)	< 0.05	99.71 (99.70–99.72)	
Non Asia	6	0.83 (0.72–0.90)	< 0.05	99.23 (99.09–99.36)		0.91 (0.82–0.96)	< 0.05	99.40 (99.31–99.50)	

^a^. *P*-Value for heterogeneity within each subgroup

^b^. *P*-Value for heterogeneity between subgroups with meta-regression analysis

### Quality assessment

**Fig. 5:**
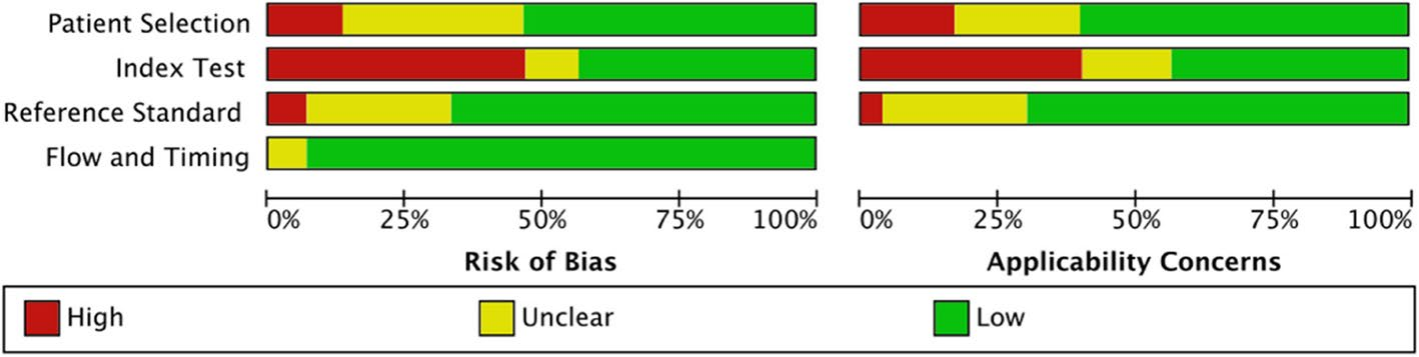
Risk of bias and applicability concerns summary about each QUADAS-AI domain presented as percentages across the 30 included studies

**Fig. 6:**
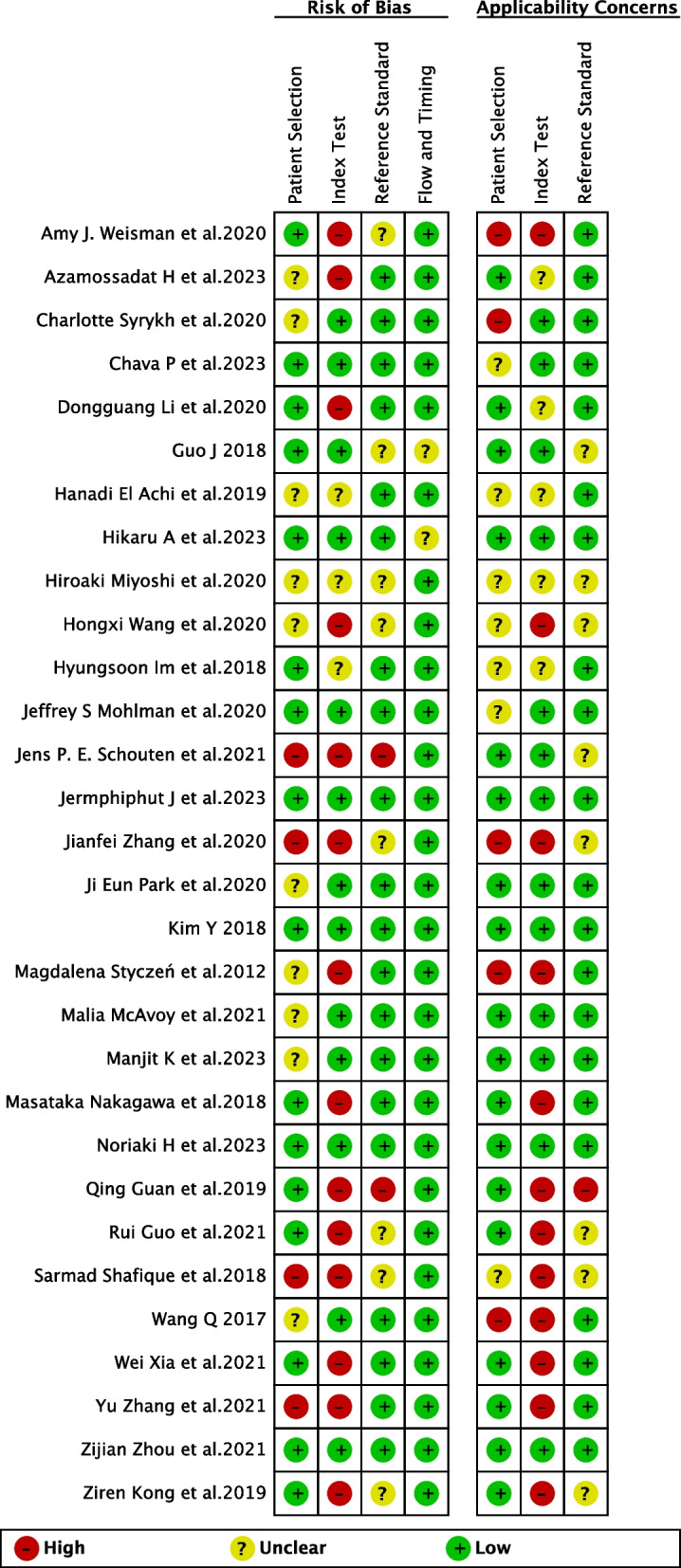
Detailed assessment for each item based on the domain of risk of bias and concern of applicability across the 30 included studies

The quality of included studies was summarized in Fig. 5 by using the QUADAS-AI tool. A detailed assessment for each item based on the domain of risk of bias and concern of applicability has also been provided as Fig. 6. For the subject selection domain of risk of bias, fourteen studies were considered a high or unclear risk of bias due to unreported rational and breakdown of training/validation/test sets, derived from open-source datasets, or not performing image pre-processing. For the index test domain, seventeen studies were considered high or at unclear risk of bias due to not performing external verification, whereas the others were considered at low risk of bias. For the reference standard domain, ten studies were considered an unclear risk of bias due to incorrect classification of target condition.

### Subgroup meta-analyses

Considering the stage of development of the algorithm and the difference in nature, we categorized them into ML and DL algorithms and did a sub-analysis. The results demonstrated a pooled SE of 86% (95% CI: 80–90%) for ML and 93% (95% CI: 88–95%) for DL, and a pooled SP of 94% (95% CI: 92–96%) for ML and 92% (95% CI: 87–95%) for DL. Additionally, six studies adopted transfer learning and ten studies did not. The pooled SE for studies that used transfer learning was 88% (80–93%), and 85 (80–89%) for studies that did not. The SP was 95% (92–97%) and 91% (88–93%), respectively.

Three studies presented the diagnostic accuracy between AI algorithms and human clinicians in the same dataset. The pooled SE was 91% (86–94%) for AI algorithms, and human clinicians had 70% (65–75%). The pooled SP was 96% (93–97%) for AI algorithms, and 86% (82–89%) for human clinicians.

Five studies had sample sizes above 200, and eleven studies used samples that were less than 200. For sample sizes under 200 and over 200, respectively, the pooled SE was 88% (84–92%) and 86% (78–91%), and the SP was 91% (87–94%) and 95% (92–97%).

Ten studies were geographically distributed in Asia and six studies were geographically distributed outside Asia. The pooled SE among studies in Asia was 88% (83–91%), whereas non-Asian studies exhibited a SE of 83% (72–90%). The pooled SP was 94% (92–96%) for studies in Asia, and 91% (82–96%) in non-Asian studies.

## Discussion

To our knowledge, this is the first systematic review and meta-analysis on the diagnostic accuracy of AI in lymphoma using medical imaging. After careful selection of studies with full reporting of diagnostic performance, we found that AI algorithms could be used for the detection of lymphoma using medical imaging with an SE of 87% and SP of 94%. We were strictly in line with the guidelines for diagnostic reviews, and conducted a comprehensive literature search in both medical databases, engineering and technology databases to ensure the rigor of the study. More importantly, we assessed study quality using an adapted QUADAS-AI assessment tool, which provides researchers with a specific framework to evaluate the risk of bias and applicability of AI-centered diagnostic test accuracy.

Although our results were largely consistent with previous research, confirming the worries that premier journals have recently raised [5, 44–46], none of the previous studies were done specifically on lymphoma. To fulfil this research gap, we strive to identify the best available AI algorithm and then develop it to enhance detection of lymphoma, and to reduce the number of false positives and false negatives beyond that which is humanly possible. Our findings revealed that AI algorithms exhibit commendable performance in detecting lymphoma. Our pooled results demonstrated an AUC of 97%, aligning closely with the performance of established conventional diagnostic methods for lymphoma. Notably, this performance was comparable to emerging radiation-free imaging techniques, such as whole-body magnetic resonance imaging (WB-MRI), which yielded an AUC of 96% (95% CI, 91–100%), and the current reference standard, 18F-fluorodeoxyglucose positron emission tomography/computed tomography (18F-FDG PET/CT), with an AUC of 87% (95% CI, 72–97%) [47]. Additionally, the SE and SP of AI algorithms surpassed those of the basic method of CT, with SE = 81% and SP = 41% [48]. However, the comparison between AI models and existing modalities was inconsistent across studies, potentially attributed to the diverse spectrum of lymphoma subtypes, variations in modality protocols and image interpretation methods, and differences in reference standards [49].

Similar to previous research in the field of image-based AI diagnostics for cancers [5, 50, 51], we observed statistically significant heterogeneity among the included studies, which makes it difficult to generalize our results with larger sample sizes or in other countries. Therefore, we conducted rigorous subgroup analyses and meta-regression for different sample sizes, various algorithms applied, geographical distribution and Al algorithms-assisted clinicians versus pure clinicians. Contrary to earlier findings [52], our results displayed that studies with smaller sample sizes and conducted in Asian regions had higher SE compared with other studies. Significant between-study heterogeneity emerged within the comparison of Al-assisted clinicians and pure clinicians. Despite this, other sources of heterogeneity could not be explained in the results, potentially attributed to the broad nature of our review and the relatively limited number of studies included.

Unlike ML, DL is a young subfield of AI based on artificial neural networks, which are known to have the capabilities to automatically extract characteristic features from images [53]. Moreover, it offers significant advantages over traditional ML methods in the early detection and diagnostic accuracy of lymphoma, including higher diagnostic accuracy [8, 14], more efficient image analysis [13], and the greater ability to handle complex morphologic patterns in lymphoma accurately [1]. Most included studies in this review investigating the use of AI in lymphoma detection employed DL (*n* = 18), with only six studies using ML. For leukemia diagnosis, the convolutional neural networks (CNN) of DL have been used, e.g., to distinguish between cases with favourable and poor prognosis of chronic myeloid leukemia [54], or to recognize blast cells in acute myeloid leukemia [55]. However, it requires far more data and computational power than ML methods, and is more prone to overfitting. Some included studies that used data augmentation methods adopting affine image transformation strategies such as rotation, translation, and flipping, to make up for data deficiencies [13, 26]. The pooled SE using ML methods was higher compared with studies using DL methods (93% VS 86%), while equivalent SP was observed between these two methods (92% VS 94%). We also discovered that AI models using transfer learning had greater SE (88% VS 85%) and SP (95% VS 91%) than models that did not. Transfer learning refers to the reuse of a pre-trained model on a new task. In transfer learning, a machine exploits the knowledge gained from a previous task to improve generalization about another. Therefore, various studies have highlighted the advantages of transfer learning over traditional AI algorithms including accelerated learning speed, reduced data requirements, enhanced diagnostic accuracy, optimal resource utilization, and improved performance in early detection and diagnostic precision of lymphoma [13, 56]. McAvoy et al. [20]. also reported that implemented transfer learning with a high-performing CNN architecture is able to classify GBM and PCNSL with high accuracy (91–92%). Within this review, no significant differences were observed between studies employing transfer learning and those that did not, as well as studies using ML or DL models, potentially indicating limitations stemming from the restricted size of datasets examined in these studies.

Evidence also suggested that AI algorithms had superior SE (91%) and SP (96%), which manifested better performance than independent detection by human clinicians (70 and 86%). Moreover, these differences were the major source of heterogeneity in the meta-regression analysis. Though AI offers certain advantages over physician diagnosis evidenced by faster image processing rates and continuous work, it does not attach importance to all the information that physicians rely on when evaluating a complicated examination. Of the included studies, only three compared the performance of integrating AI with clinicians and pure algorithms, which also restricts our ability to extrapolate the diagnostic benefit of these algorithms in medical care delivery. In the future, the AI versus physicians dichotomy is no longer advantageous, and an AI-physician combination would drive developments in this field and largely reduce the burden of the healthcare system. On one hand, future non-trivial applications of AI in medical settings may need physicians to combine pieces of demographic information with image data, optimize the integration of clinical workflow patterns and establish cloud-sharing platforms to increase the availability of annotated datasets. On the other, AI could perhaps serve as a cost-effective replacement diagnostic tool or an initial method of risk categorization to improve workflow efficiency and diagnostic accuracy of physicians.

Though our review suggests a more promising future of AI upon current literature, some critical issues in methodology needed to be interpreted with caution:

Firstly, only one prospective study was identified, and it did not provide a contingency table for meta-analysis. In addition, twelve studies used data from open-accessed databases or non-target medical records, and only eleven were conducted in real clinical environments (e.g., hospitals and medical centers). This is well known that prospective studies would provide more favorable evidence [57], and retrospective studies with data sources in silicon might not include applicable population characteristics or appropriate proportions of minority groups. Additionally, the ground truth labels in open-assessed databases were mostly derived from data collected for other purposes, and the criteria for the presence or absence of disease were often poorly defined [58]. The reporting around handling of missing information in these datasets was also poor across all studies. Therefore, the developed models might lack generalizability, and studies utilizing these databases may be considered as studies for proof-of-concept technical feasibility instead of real-world experiments evaluating the clinical utility of AI algorithms.

Second, in this review, only six studies performed external validation. For internal validation, three studies adopted the approach of randomly splitting, and twelve used cross-validation methods. The performance judged by in-sample homogeneous datasets may potentially lead to uncertainty around the estimates of diagnostic performance, therefore it is vital to validate the performance using data from a different organization to increase the generalizability of the model. Additionally, only five studies excluded poor-quality images and none of them were quality controlled for the ground truth labels. This may render the AI algorithms vulnerable to mistakes and unidentified biases [59].

Third, though no publication bias was observed in this review, we must admit that the researcher-based reporting bias could also lead to overestimating the accuracy of AI. Some related methodological guides have recently been published [60–62], while the disease-specific AI guidelines were not presented. Since researchers tend to selectively report favorable results, the bias might be likely to skew the dataset and add complexity to the overall appraisal of AI algorithms in lymphoma and its comparison with clinicians.

Fourth, the majority of studies included were performed in the absence of AI-specific quality assessment criteria. Ten studies were considered to have low risk in more than three evaluation domains, while nine studies were considered high risk under the AI-specific risk of bias tool. Previous studies most commonly used the quality assessment of diagnostic accuracy studies (QUADAS-2) tool to assess bias and applicability encouraged by current PRISMA 2020 guidance [63], which does not address the particular terminology that arises from AI diagnostic test studies. Furthermore, it did not take into account other challenges that arise in AI research, such as algorithm validation and data pre-processing. QUADAS-AI provided us with specific instructions to evaluate these aspects [16], which is a strength of our systematic review and will help guide future relevant studies. However, it still faces several challenges [16, 64] including incomplete uptake, lack of a formal quality assessment tool, unclear methodological interpretation (e.g., validation types and comparison to human performance), unstandardized nomenclature (e.g., inconsistent definitions of terms like validation), heterogeneity of outcome measures, scoring difficulties (e.g.,uninterpretable/intermediate test results), and applicability issues. Since most of the relevant studies were more often designed or conducted prior to this guideline, we accepted the low quality of some of the studies and the heterogeneity between the included studies.

This meta-analysis has some limitations that merit consideration. Firstly, a relatively small number of studies were available for inclusion, which could have skewed diagnostic performance estimates. Additionally, the restricted number of studies addressing diagnostic accuracy in each subgroup, such as specific lymphoma subtypes and medical imaging modalities, prevented a comprehensive assessment of potential sources of heterogeneity [65, 66]. Consequently, the generalizability of our conclusions to diverse lymphoma subtypes and varied medical imaging modalities, particularly without the integration of AI models at this current stage, could be limited. Secondly, we did not conduct a quality assessment for transparency since current diagnostic accuracy reporting standards (STARD-2015) [67] is not fully applicable to the specifics and nuances of AI research. Thirdly, several included studies have methodological deficiencies or are poorly reported, which may need to be interpreted with caution. Furthermore, the wide range of imaging technology, patient populations, pathologies, study designs and AI models used may have affected the estimation of diagnostic accuracy of AI algorithms. Finally, this study only evaluated studies reporting the diagnostic performance of AI using medical image, which is difficult to extend to the impact of AI on patient treatment and outcomes.

To further improve the performance of AI algorithms in detecting lymphoma, based on the aforementioned analysis, focused efforts are required in the domains of robust designs and high-quality reporting. To be specific, firstly, a concerted emphasis should be directed towards fostering an augmented landscape of multi-center prospective studies and expansive open-access databases. Such endeavors can facilitate the exploration of various ethnicities, hospital-specific variables, and other nuanced population distributions to authenticate the reproducibility and clinical relevance of the AI model. Therefore, we suggest the establishment of interconnected networks between medical institutions, fostering unified standards for data acquisition, labeling procedures and imaging protocols to enable external validation in professional environments. Additionally, we also call for prospective registration of diagnostic accuracy studies, integrating a priori analysis plan, which would help improve the transparency and objectivity of reporting studies. Second, we would encourage AI researchers in medical imaging to report studies that do not reject the null hypothesis, which might improve both the impartiality and clarity of studies that intend to evaluate the clinical performance of AI algorithms in the future. Finally, though time-consuming and difficult [68], the development of “customized” AI models tailored to specific domains, such as lymphoma, head and neck cancer [69], or brain MRI [70], emerges as a pertinent suggestion. This tailored approach, encompassing meticulous preparations such as feature engineering and AI architecture, alongside intricate calculation procedures like segmentation and transfer learning, could yield substantial benefits for both patients and healthcare systems in clinical application.

## Conclusions

This systematic review and meta-analysis appraised the quality of current literature and concluded that AI techniques may be used for lymphoma diagnosis using medical images. However, it should be acknowledged that these findings are assumed in the presence of poor design, methods and reporting of studies. More high-quality studies on the AI application in the field of lymphoma diagnosis with adaption to the clinical practice and standardized research routines are needed.

### Supplementary Information

**Additional file 1.** Search terms and search strategy.

**Additional file 2.** PRISMA checklist.

## Data Availability

The search strategy and extracted data contributing to the meta-analysis is available in the supplement document; any additional data are available from the corresponding author upon reasonable request.

## Abbreviations

HL: Hodgkin Lymphoma
NHL: Non-Hodgkin Lymphoma
DLBCL: Diffuse Large B-cell Lymphoma
PCNSL: Primary Central Nervous System Lymphoma
ALL: Acute Lymphoblastic Leukemia
ENKTL: Extranodal NK/T Cell Lymphoma
MRI: Magnetic Resonance Imaging
WSI: Whole Slide Image
PET/CT: Positron Emission Tomography / Computedtomography
FL: Follicular Lymphoma
FH: Follicular hyperplasia
AI: Artificial intelligence
PRISMA: Preferred Reporting Items for Systematic reviews and Meta-analyses
QUADAS-2: Quality Assessment of Diagnostic Accuracy Studies 2
SROC: Summary Receiver Operating Characteristic Curves
DL: Deep Learning
CI: Confidence Intervals
SE: Sensitivity
SP: Specificity
