# Supplementary material for: Artificial intelligence performance in detecting lymphoma from medical imaging: a systematic review and meta-analysis
Source: BMC Med Inform Decis Mak. 2024 Jan 8;24:13. doi: 10.1186/s12911-023-02397-9 (PMC10775443; doi:10.1186/s12911-023-02397-9)
Supplement: Supplementary file 1 — Additional file 1. Search terms and search strategy. [file 12911_2023_2397_MOESM1_ESM.docx]

**Supplement Document**

**Artificial intelligence performance in detecting lymphoma from medical imaging: A systematic review and meta-analysis**

**Supplementary Material 1: Search terms and search strategy**

**Medline**

1. "lymph*".ab,hw,kf,ti,tw.
2. "cancer".ab,hw,kf,ti,tw.
3. "*neoplasms*".ab,hw,kf,ti,nm.
4. 2 or 3
5. ("*sensitivity and specificity*" or "*roc curve*" or "*Calibration*" or "*area under the curve*" or roc or auc or "goodness of fit" on performance or calibrat* or accuracy* or sensitivity or specificity).ti,ab.
6. 1 And 4 And 5
7. "image".ab,hw,kf,ti,tw.
8. (("*artificial intelligence*" or "*machine learning*" or "*deep learning*" or "*neural network*") not (decision tree or random forest or nearest neighbo$ or naive bayes or "*vector machine")).ab,hw,kf,ti,nm.
9. 6 and 7 and 8

**Embase**

#1 'artificial intelligence'/exp OR 'machine learning'/exp OR 'deep learning'/exp OR 'artificial neural network'/exp

#2 'decision tree'/exp OR 'random forest'/exp OR 'bayesian learning'/exp OR 'support vector machine'/exp

#3 'lymph*'/exp

#4 'malignant neoplasm'/exp OR 'cancer'/exp

#5 'performance'/exp OR 'sensitivity and specificity'/exp OR 'diagnostic accuracy'/exp OR 'area under the curve'/exp OR ‘goodness of fit' OR 'calibrat*'/exp

#6 'image*'/exp

#7 #1 NOT #2

#8 #3 AND #4 AND #5 AND #6 AND #7

**IEEE**

((((((((No Keywords Specified))) AND ((Full Text & Metadata:artificial intelligence) OR (Full Text & Metadata:machine learning) OR (Full Text & Metadata:deep learning) OR (Full Text & Metadata:neural network))) NOT ((Full Text & Metadata:decision tree) OR (Full Text & Metadata:random forest) OR (Full Text & Metadata:naive bayes) OR (Full Text & Metadata:nearest neighbor) OR (Full Text & Metadata:vector machine))) AND ((Full Text & Metadata:cancer) OR (Full Text & Metadata:neoplasms) OR (Full Text & Metadata:tumor) refined by:Year:1884-2023 )) AND ((Full Text & Metadata:lymph*))) AND ((Full Text & Metadata:performance) OR (Full Text & Metadata:sensitivity) OR (Full Text & Metadata:specificity) OR (Full Text & Metadata:accuracy) OR (Full Text & Metadata:area under the curve) OR (Full Text & Metadata:AUC) OR (Full Text & Metadata:ROC) OR (Full Text & Metadata:goodness of fit) OR (Full Text & Metadata:calibrat))) AND ((Full Text & Metadata:image))

**Cochrane**

#1 - (artificial intelligence):ti,ab,kw OR (machine learning):ti,ab,kw OR (deep learning):ti,ab,kw OR (neural network):ti,ab,kw

#2 - (lymph*):ti,ab,kw

#3 - (Cancer):ti,ab,kw OR (Neoplasms):ti,ab,kw

#4 - (Sensitivity and Specificity):ti,ab,kw OR (Accuracy):ti,ab,kw OR (Area Under Curve):ti,ab,kw OR (Sensitivity):ti,ab,kw OR (Specificity):ti,ab,kw

#5 - #1 AND #2 AND #3 AND #4
